# Yttria-Stabilized Zirconia Deposited Using Suspension Plasma Spraying: A Comparative Framework with Electron Beam Physical Vapor Deposition and Air Plasma Spraying

**DOI:** 10.1021/acsami.6c05211

**Published:** 2026-06-12

**Authors:** Vikram Hastak, Kah Leng, Siddharth Lokachari, Nicholas Curry, Gyaneshwara Brewster, Andy Norton, Tanvir Hussain

**Affiliations:** 1 Centre of Excellence in Coatings and Surface Engineering, Faculty of Engineering, 6123 University of Nottingham, Nottingham NG7 2RD, U.K.; 2 Thermal Spray Innovations, Salzburg 5662, Austria; § 2934 Rolls-Royce Plc, Derby DE24 8BJ, U.K.

**Keywords:** yttria-stabilized zirconia, thermal barrier coatings, suspension plasma spraying, thermal cycling, CMAS resistance

## Abstract

Emerging technological advancements are aimed at improving the operating temperatures of gas turbine engines to improve their overall efficiency. This has triggered the development of yttria-stabilized zirconia-based thermal barrier coatings (YSZ TBC) with microstructures designed for enhanced performance achieved by optimizing process parameters and deposition techniques. The present study aims to evaluate the performance of YSZ columnar structure deposited using suspension plasma spraying (SPS) on Pt–Al bond-coated CMSX-4 single-crystal nickel-based superalloy substrate, reported for the first time in the literature. The thermal cycling performance and calcium–magnesium-alumino-silicate (CMAS) corrosion behavior of SPS YSZ coatings were compared with YSZ deposited using electron beam physical vapor deposition (EBPVD) and air plasma spraying (APS) techniques. It was observed that SPS YSZ coatings showed the highest thermal cycling resilience when compared to EBPVD and APS. In addition to this, the CMAS penetration was also substantially lower in SPS YSZ coatings compared with EBPVD and APS coatings. Unlike SPS and EBPVD coatings, APS coatings also showed signs of tetragonal-to-monoclinic phase transitions in some regions away from the top surface. These observations lay the baseline for the potential development of optimized microstructures using the SPS deposition technique with improved performance relevant to the TBCs.

## Graphical abstract

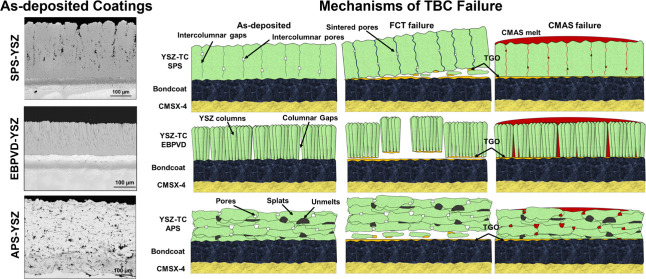

## 1 Introduction

Over the past several decades, thermal barrier coatings (TBCs) have been extensively used to enhance the performance of gas turbine engines.^1–4^ These TBCs serve as a protective layer to reduce the temperature of the metallic counterparts and increase the engine efficiency by enabling increased turbine inlet temperatures.^5,6^ TBCs are manufactured by first depositing a bond coat on the metallic component, which provides oxidation protection and enhances adhesion of the topcoat. This is then followed by the deposition of a thermal barrier topcoat, which is typically zirconia partially stabilized with ∼7–8 wt % of yttria (YSZ), owing to its high temperature stability, low thermal conductivity, coefficient of thermal expansion compatible with Ni-based superalloy, and higher fracture toughness.^7,8^

Most commercial TBCs are deposited via atmospheric plasma spraying (APS) and electron beam physical vapor deposition (EBPVD) techniques. The APS deposition process provides ease of application and cost-effectiveness, giving rise to splat morphology. In contrast, EBPVD, an expensive high-vacuum process, is used for the development of columnar microstructures with high in-plane strain tolerance (critical in-plain strain at spallation for EBPVD YSZ coatings is reported to be on the order of ∼3 × 10^–3^–6 × 10^–3^ (≈0.3–0.6%), however, conventional APS coatings exhibit significantly lower critical strain values on the order of ∼1–2 × 10^–3^ (≈0.1–0.2%)^1,9,10^ ). These TBC’s resilience to harsh temperature gradients, oxidative and corrosive environments is frequently assessed using a variety of testing techniques.^11,12^ The intrinsic failures in TBCs include coating delamination, formation of thermally grown oxides during service, phase transformations, and crack propagation. Apart from this, TBCs are also susceptible to extrinsic failures such as foreign object damage/erosion and deterioration due to molten dust (calcium–magnesium-alumino-silicate; CMAS) attack.^11,13^

There have been tremendous technological and scientific advancements in TBCs toward the development of new materials with enhanced properties and engineering-optimized microstructures to increase the lifespan of the coatings using various deposition techniques. The effort to modify the microstructures is directed toward developing low thermal conductivity, high strain tolerance, and thermally stable thermal barrier topcoats.^14^ The performance of TBCs indeed depends upon the microstructure of the topcoat. For instance, the thermal cycling lifetime of APS-TBCs is often lower compared to EBPVD-TBCs because of the strain energy-induced early spallation in the former; however, the thermal conductivity is reported to be higher in the latter. The thermal conductivity of EBPVD is reported to be around ∼1.4–1.9 W/mK, which is higher than that of APS YSZ coatings exhibiting ∼0.5–1 W/mK thermal conductivity.^9,15–19^ Suspension plasma spraying (SPS) is a widely utilized technique that offers a broad range of parameters, which can be optimized to achieve an EBPVD-like columnar structure with lower thermal conductivity.^20^ In SPS, fine submicron powders are deposited onto the substrate by the evaporation of fine droplets formed as a result of the atomization of powder suspensions when they are injected into the plasma plume.^21^ The broad range of parameters that can be altered and optimized includes plasma gas composition, gas flow rate, current, net power, and resulting plasma enthalpy, as well as suspension feed rate, suspension concentration, solvent type, particle size, stand-off distance, and substrate temperature. These parameters collectively control the coating microstructure and, consequently, the coating performance. Various authors have reported microstructures ranging from lamellar to columnar and dense vertically cracked (DVC) structures obtained using SPS that exhibit promising and more favorable thermal cycling lifetimes as compared to APS and EBPVD-TBCs;^18,20,22–25^ however, EBPVD-like columnar microstructures could be detrimental against CMAS as it acts as a pathway for the infiltration of CMAS melts leading to coating failure.^26,27^ CMAS infiltration depth is governed by several factors, including the viscosity and surface tension of the molten CMAS, as well as the exposure temperature and infiltration duration. For EBPVD coatings, Naraparaju et al.^28,29^ reported that infiltration within the feathery columnar arms increased significantly with time at 1225 °C, reaching approximately ∼10%, ∼50%, and ∼90% of the coating thickness after 10, 20, and 30 h, respectively. For SPS coatings, Lokachari et al.^30^ reported CMAS infiltration depths of ∼190–270 μm after 5 min at 1250 °C. In contrast, significantly deeper penetration has been observed in APS coatings, where infiltration depths of up to ∼500 μm have been reported in service-exposed TBCs in an actual engine environment.^31^

The present study reports, for the first time, a detailed comparison of the performance of 8YSZ deposited on CMSX-4 single-crystal Ni-based superalloy substrate using three different and most widely used deposition techniques, viz. SPS, EBPVD, and APS. The performance was evaluated by exposing the coatings together against two widely used failure tests in TBC, namely, thermal cycling and CMAS corrosion. The investigation of the underlying mechanisms and behavior of failure was then carried out using scanning electron microscopy (SEM), X-ray diffraction (XRD), and Raman spectroscopy. The aim was to understand the mechanisms of various properties required for TBCs manufactured through different state-of-the-art manufacturing routes.

## 2 Experimental Methodology

### 2.1 Coating Materials and Manufacturing

An 8 wt % YSZ ethanol-based suspension was supplied by Treibacher Industrie AG (Althofen, Austria), which had 25 wt % solid loading of YSZ with a *D*_50_ particle size of 500 nm. The suspension was agitated by placing it on a roller (Capco, Suffolk, UK) at 50 rpm for 1 h prior to the coating deposition to ensure mitigation of agglomerated particles sedimented at the bottom and obtain a homogeneous suspension with uniformly distributed YSZ particles.

Single-crystal nickel-based CMSX-4 superalloy disks (22.6 mm diameter x 5 mm thick) were provided with a platinum aluminide (PtAl) bondcoat for SPS and EBPVD depositions and an air plasma-sprayed (APS) CoNiCrAlY (Amdry 995C) bondcoat for APS 8YSZ deposition. CMSX-4 is a second-generation nickel-based single-crystal (SX) superalloy widely used for high-pressure turbine blades and vanes in aircraft engines due to its exceptionally high-temperature strength, creep resistance, and stability at high temperature. It contains 3 wt % rhenium (Re), 6.5% Cr, 5.6% Al, 9.6% Co, 6.4% W, 0.6% Mo, 0.1% Hf, 6.5% Ta, and Ni (balance) designed by Cannon-Muskegon Corporation.^32^ Air plasma-sprayed (APS) and electron beam physical vapor (EBPVD)-deposited YSZ TBCs were supplied by Rolls-Royce.

The SPS YSZ topcoat was deposited on the bond coat using Axial III (Northwest Mettech Corp., Surrey, BC, Canada), a trianode/tricathode high-power DC plasma torch system with an exit nozzle diameter of 0.375 in. and a suspension injector of 250 μm. The bond-coated substrates were preheated for 10 cycles, following which the YSZ suspension feed was introduced. The topcoat deposition consisted of 45 passes to achieve ∼250 μm thickness. The coatings with columnar microstructure were developed by optimizing the spray parameters reported elsewhere,^22^ which include gas flow rate, gas composition (Ar:N_2_:H_2_), scan speed, and the stand-off distance, thus controlling the temperature and the velocity of the spray particles impacting the substrate. The SPS YSZ coating deposition parameters include gas composition of 44/28/28 (Ar/N_2_/H_2_ by vol %) with a total gas flow rate of 300 L/min, 70 mL/min suspension feed rate, 200 A current, 100 mm stand-off distance, and a robot raster scan speed of 1600 mm/s. The parameters here were slightly changed, considering the effect of a smoother surface of the bondcoat by lowering the feed rate and increasing the stand-off distance, thus reducing the particle velocity and temperature, which leads to a greater tendency to form columnar and strain-compliant porous microstructure.^23,30,33^ SPS parameter optimization reported by Lokachari et al.^30^ was carried out on HVOF-sprayed CoNiCrAlY bond coat. These parameters were used as a starting point but were reoptimized to achieve a similar columnar microstructure on a smoother Pt–Al bond coat, where different spreading and growth dynamics necessitate modified deposition conditions. With a lower feed rate, atomization of the suspension becomes more dominant, resulting in the formation of finer molten droplets. At a higher stand-off distance (SOD), these finer droplets have more time to follow the plasma drag, increasing their horizontal velocity component. This enhanced lateral motion allows them to be more effectively captured by the peaks of a smooth bond coat surface.^34^ The EBPVD and APS samples were provided by our industrial collaborators, and the deposition parameters are commercially sensitive and are not disclosed. Nonetheless, these EBPVD and APS coatings have been used extensively in literature for various performance studies.^35–37^

### 2.2 Coatings Performance Tests

#### 2.2.1 Furnace Cycling Test (FCT)

The furnace cycling test was performed in a bottom-loading isothermal furnace (CM Furnaces Inc., Bloomfield, New Jersey, USA). The furnace cycling involved three steps: the samples were exposed to a temperature of 1135 °C at a high heating rate (within a span of ∼10 min), isothermally held for 45 min, and then cooled by employing forced air cooling with a pressure of ∼1 bar (∼30 min cooling time). A total of 9 samples, comprising three coating specimens from each of three different deposition techniques (SPS, EBPVD, and APS), were placed on a refractory platform and were exposed to furnace cycling degradation until failure.

The coating’s failure criteria were based on the extent of their spallation emerging over ∼20% of the surface of the topcoat. This was examined by using a high-definition Webcam (Logitech C930e, Lausanne, Switzerland) that captures images at 1 min intervals.

#### 2.2.2 CMAS Exposure Test

CMAS with a nominal composition of 35CaO-10MgO-7Al_2_O_3_-48SiO_2_ (in mol %; Oerlikon Metco, Cheshire, U.K.) was mixed with DI water in a 1:9 ratio to form a solution. The CMAS solution was constantly stirred using an Isotemp hot plate (Fisher Scientific, Loughborough, UK) with a magnetic stirrer. The agitated CMAS solution was then sprayed evenly on the coatings using an airbrush kit while heating them at 100 °C to evaporate the DI water. The CMAS loading was selected as 15 mg/cm^2^ as per the widely accepted and established protocol. The concentration was measured by taking sample weights pre- and post-CMAS deposition. Once the desired CMAS loading was attained, the samples were exposed to a temperature of 1250 °C for 30 min at a heating and cooling rate of 10 °C in a box furnace (Elite Thermal Systems Ltd., Leicester, U.K.).

### 2.3 Sample Preparation

The coatings after FCT and CMAS tests were impregnated first in an epoxy-hardener matrix under a vacuum and cured at room temperature for 12 h. These were then sectioned using a diamond cutoff wheel (Metprep, UK) in a precision cutter supplied by Metprep, UK. The cut samples were then double cold-mounted and ground using SiC grinding papers, followed by polishing using diamond pads. The as-deposited samples were sectioned and cold-mounted to obtain preliminary microstructures.

### 2.4 Characterization

#### 2.4.1 Scanning Electron Microscopy and X-ray Diffraction

The cross-sectional microstructures and compositional information were examined by using a scanning electron microscope, Jeol JSM IT-200 SME (JEOL Ltd., Tokyo, Japan), equipped with a tungsten electron source. Both the as-deposited and tested samples were analyzed in both secondary electron (SE) and backscattered electron (BSE) modes at an accelerating voltage of 15 kV and a working distance of 10 mm to understand the underlying mechanism of failure. Three images for each sample at 200× magnification (512 × 640 μm; BSE) were taken for measuring the thickness and porosity. Twenty vertical lines were drawn in each image for measuring the average thickness, and the porosity was measured using a thresholding technique. The phase analysis was carried out using X-ray diffraction, Bruker D8 Advance with the DaVinci system. XRD diffractograms were recorded in the range 10° to 90° 2θ with Cu Kα radiation (1.54 wavelength) with a step size of 0.01° and a time per step of 1 s in Bragg–Brentano geometry. Prior to XRD analysis, K_α2_ signals were stripped from the raw XRD pattern in Philip’s X-Pert High Score Plus software using the Rachinger method.

#### 2.4.2 Raman Spectroscopy

Raman spectra of the CMAS exposed coatings were obtained using a HORIBA LabRAM HR Raman microscope (Horiba Jobin YVON, Japan) equipped with a Synapse detector and an automated xyz stage (Marzhauser, Germany) using a 532 nm laser. Si (100) reference sample with characteristic band at 520.7 cm^–1^ was used to calibrate the instrument. The 49 points spectra were collected over a region covering the top ∼80 μm of the topcoat using a 1800 grooves/mm grating, a 200 μm pinhole, and a 50× objective. The power, acquisition time, and number of accumulations were optimized to achieve a better signal-to-noise ratio. The spectra were then analyzed using LabSpec 6 software (Horiba Jobin YVON, Japan) and Origin Pro (OriginLab Corp., Northampton) software.

#### 2.4.3 Microhardness

The microhardness of SPS, APS, and EBPVD YSZ coatings was measured using a Wilson VH3300 Vickers microhardness indenter coupled with an optical microscope. The microhardness tests were performed on polished cross-sectioned coatings with a load of 100 gf (HV0.1; 0.98 N) and dwell time of 10 s on five different regions. The microhardness values obtained from five indents were then averaged to obtain the final microhardness value of each coating.

## 3 Results

### 3.1 Preliminary Characterization

The as-deposited microstructures of SPS, EBPVD, and APS YSZ topcoats are shown in Figure 1. It was evident from the cross-sectional micrographs that both SPS and EBPVD coatings had columnar YSZ deposited onto the bondcoat. The idea behind developing a columnar structure using SPS is to have improved thermal cycling lifetime, which is favored in strain-compliant EBPVD columns. The reported improvement in thermal cycling lifetime is evaluated with respect to other coatings exhibiting noncolumnar (conventional porous, dense vertically cracked) atmospheric plasma spray coating microstructures.^38–40^ This can be achieved by controlling the plasma enthalpy, suspension properties (solid loading, solvent, injection rate), stand-off distance, and substrate temperature, which together govern droplet size, melting state, and columnar deposition.^41^ While EBPVD coatings showed columnar features with faceted tips owing to the shadowing effect (preferential orientation), the SPS coatings had cauliflower-shaped columns with comparatively lower column density. The optimization of spray parameters to obtain a thermo-chemico-mechanically robust and resilient columnar YSZ coating is based on a comprehensive SPS parameter optimization strategy as reported by Lokachari et al.^30^ The APS coatings showed characteristic planar YSZ splats stacked with a stochastic presence of pores and cracks. The average column density of EBPVD YSZ coatings was measured to be 163 ± 13 columns/mm, with an average column diameter of 6 ± 0.5 μm. In contrast, SPS YSZ coatings exhibited a significantly lower column density of 19 ± 3 columns/mm and a much larger column diameter of 54 ± 7 μm.

**Figure 1:**
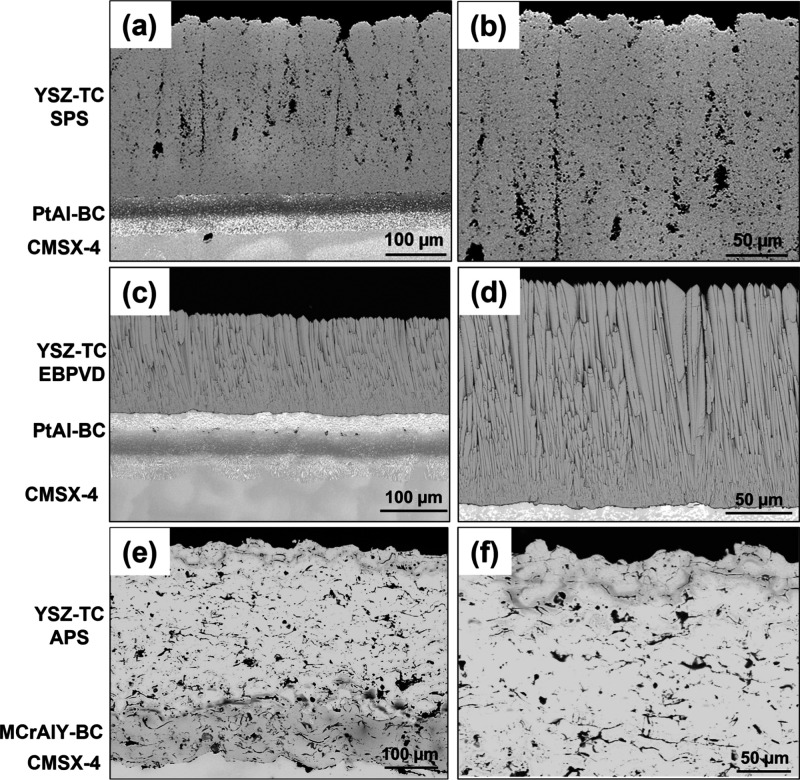
SEM cross-sectional micrographs of (a, b) SPS, (c, d) EBPVD, and (e, f) APS YSZ coatings.

The XRD of the as-deposited samples, as shown in Figure 2a matched well with the *t*′ tetragonal YSZ crystal system^42–44^ (Reference Pattern; 01–083–0113). EBPVD coatings showed columns with preferred orientation along the <110> direction concurrent with the observed faceted columnar tips.

**Figure 2:**
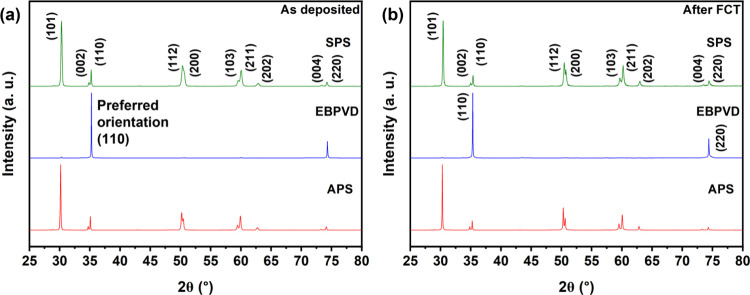
XRD of SPS, EBPVD, and APS YSZ coatings; (a) as deposited and (b) after furnace thermal cycling. XRD of the failed coatings post thermal cycling shows no evidence of phase transformations.

Table 1 shows the coating thickness and porosity measured using ImageJ. The coating thickness certainly influences both the residual stresses and those arising from differences in the coefficient of thermal expansion (CTE). However, beyond differences in measured coating thickness, it is important to note that these coatings were developed following optimization within three individual deposition techniques. Therefore, a comparison is made between the best-performing coatings obtained from each of these techniques. The microhardness value reported here is the average of multiple indentation microhardness measurements for each coating. These values were normalized for comparative purposes while putting APS, which had the lowest hardness value, as a reference. The microhardness of SPS YSZ coatings was found to be the highest among all the coatings.

**Table 1:** Coating Thickness, Porosity, and Microhardness of the Coatings^a^

sample	thickness (μm)	porosity	normalized microhardness (HV0.1)
SPS	266 ± 2	10% ± 2%	(1.31 ± 0.15)*K*
EBPVD	148 ± 4	12% ± 1%	(1.03 ± 0.16)*K*
APS	309 ± 10	10% ± 2%	(1 ± 0.13)*K*

a K is used as a hardness constant for comparison.

### 3.2 Furnace Cycling Tests

The coatings were then tested against thermal cycling. Figure 3 shows the plan view of the coatings at different stages of thermal cycling events (with N_T_ being the maximum number of thermal cycles). These images were captured periodically while taking into account the thermal cycling failure events of the various coatings. The camera view of the coatings after subsequent thermal cycling is shown in Figure S1. The number of cycles to failure was normalized for comparative analyses with respect to the maximum thermal cycling fatigue life of the coatings, which showed the best performance under the same thermal cycling conditions. It was observed that APS YSZ coatings exhibited the earliest delamination. The normalized number of cycles to failure for all the APS coatings was significantly lower than that of the other coating systems. The EBPVD YSZ coating showed an intermediate thermal cycling durability performance in terms of normalized cycles to failure. Unlike APS coatings, some portions of EBPVD coatings had not spalled when the failure criteria were met. Compared to APS and EBPVD coatings, SPS coatings achieved the highest number of cycles to failure. Similar to EBPVD coatings, SPS coatings also exhibited partial spallation post-thermal cycling.

**Figure 3:**
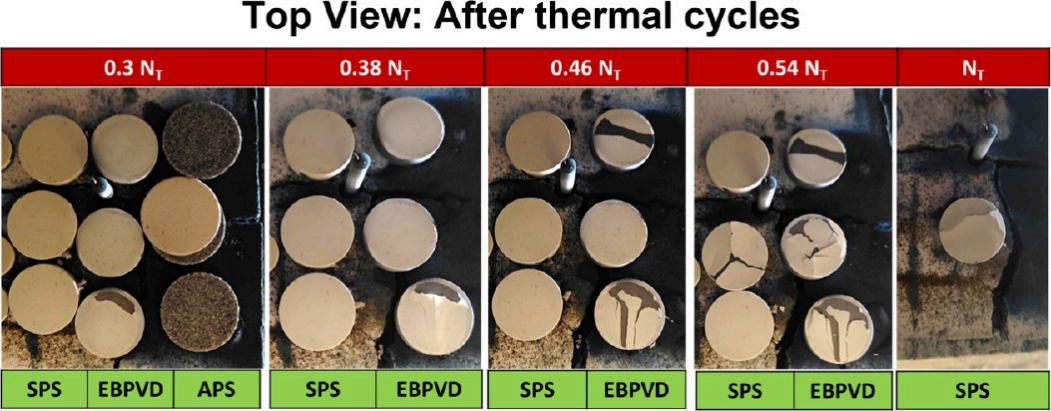
Plan view of specimens exposed to thermal cycling showing three samples of SPS, EBPVD, and APS samples. Samples were removed when failure criteria was achieved. *N*_T_ is the maximum number of thermal cycles.

The failure mechanisms observed in individual coatings are explained as follows:

#### 3.2.1 SPS FCT Failure

The mechanism of thermal shock failure leading to delamination of the SPS coatings is predominantly governed by crack initiation and propagation near the interface between the topcoat and the bond coat.^45–47^ Out of the three SPS coatings tested against thermal cycling, one survived the highest number of thermal cycles (denoted as *N*_T_). The other two failed after 0.53 and 0.71 *N*_T_ cycles. Figure 4 shows the cross-sectional micrographs of the failure mechanism in SPS YSZ TBC post *N*_T_ cycles. There was significant thermal exposure-induced sintering of columns and widening of vertical cracks after thermal cycling. As a result of thermal cycling, it is evident from the micrographs and elemental mapping that spallation predominantly occurred at the TGO-topcoat interface, within the TGO, and at the TGO-bondcoat interface. There were a few regions where the cracks from the TGO propagated into the topcoat, leading to delamination within the topcoat. The thickness of the TGO as measured using ImageJ was 5.7 ± 1.1 μm. This TGO thickness is close to the critical thickness, which is likely to be one of the possible reasons for spallation, as proposed in the literature.^25,48^ It must be noted that a significant portion of the coatings remained adhered to the bond coat even after maximum cycles (*N*_T_) of thermal exposure. The crystal structure of the failed coatings as observed using XRD corresponds to the *t’-*ZrO_2,_ similar to the as-deposited state, which suggests that there was no evident phase transformation (*t* to *m-*ZrO_2_) at the top surface of the coatings even after thermal cycling failure (Figure 2).

**Figure 4:**
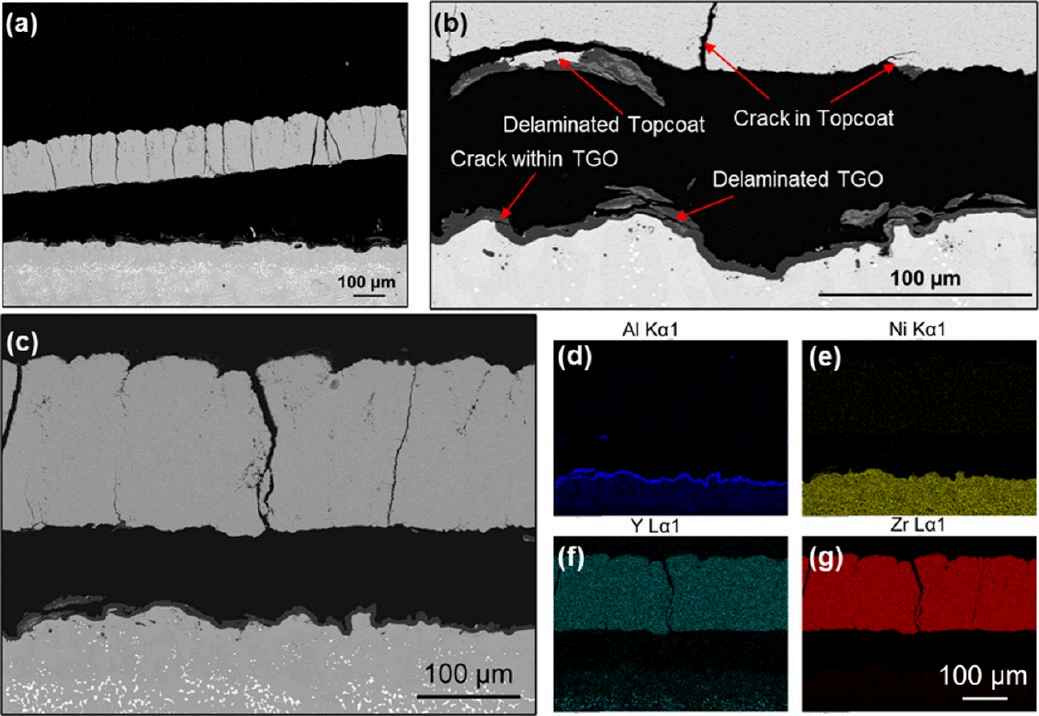
SPS YSZ TBC FCT failure; (a) cross-sectional micrographs of the failed SPS coatings post maximum (*N*_T_) thermal cycles, (b) zoomed region showings cracks and delamination of the topcoat and the bond coat, (c) EDS mapping region and corresponding element maps of (d) aluminum, (e) nickel, (f) yttrium, and (g) zirconium.

#### 3.2.2 EBPVD FCT Failure

Figure 5 shows the cross-sectional micrographs and EDS mapping after failure of the EBPVD coating post 0.54 *N*_T_ cycles. Like SPS coatings, the thermal cycling failure in EBPVD coatings was observed at the TGO-topcoat, TGO-TGO, and the TGO-bondcoat interface. The first coating failed after 0.43 *N*_T_ cycles, followed by the failure of the remaining two after 0.47 N_T_ and 0.52 *N*_T_ cycles. The thickness of the TGO as measured using ImageJ was 4.6 ± 0.9 μm.

**Figure 5:**
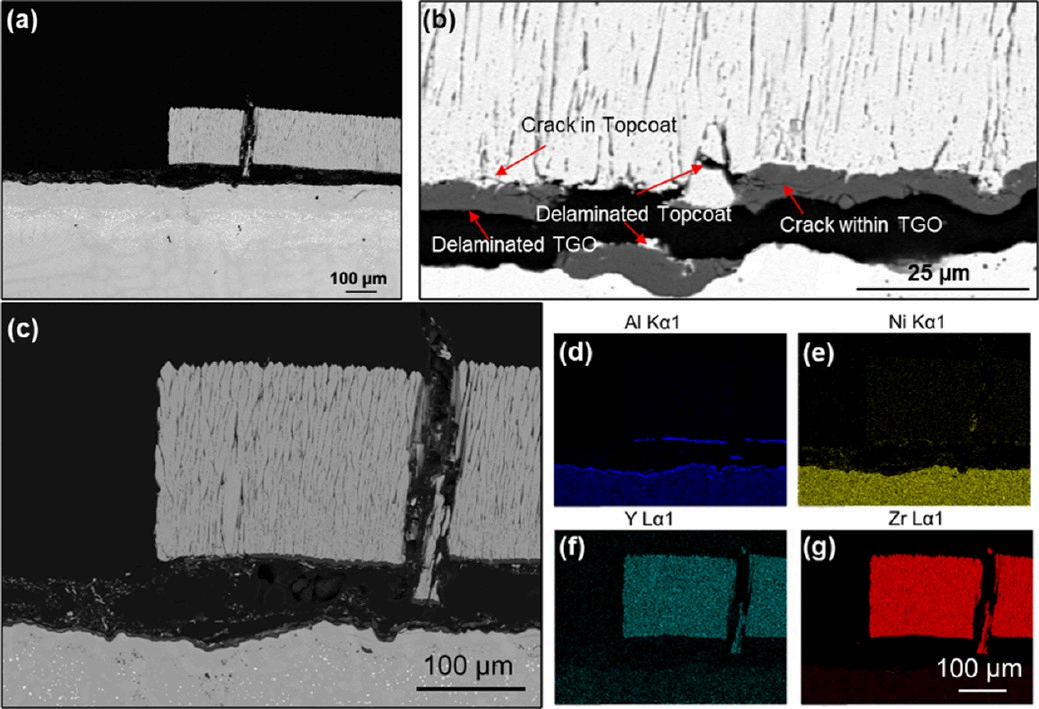
EBPVD YSZ TBC FCT failure; (a) cross-sectional micrographs of the failed EBPVD coatings post 0.54 N_T_ thermal cycles, (b) zoomed region showings cracks and delamination of the topcoat and the bond coat, (c) EDS mapping region and corresponding element maps of (d) aluminum, (e) nickel, (f) yttrium, and (g) zirconium.

The failure in the EBPVD topcoat initiated with the development of horizontal cracks at the TGO-bond coat interface, which extended toward the topcoat, leading to delamination. A massive vertical crack failure near columnar gaps was observed, which could be due to the interaction of horizontal delamination with the preexisting and growing columnar gaps. It is well-known that the thermal stresses developed due to the coefficient of thermal expansion mismatch are one of the primary causes of TBC spallation. Like SPS coatings, no martensitic *t* → *m* phase transformations, i.e., monoclinic phase formation, were evident after thermal shock as observed from the XRD of the failed coatings (Figure 2b).

#### 3.2.3 APS FCT Failure

Figure 6 shows the cross section of the failed APS topcoat post 0.3 *N*_T_ cycles. Two APS coatings failed after 0.25 *N*_T_ thermal cycles, while the third one survived until 0.27 *N*_T_ cycles.

**Figure 6:**
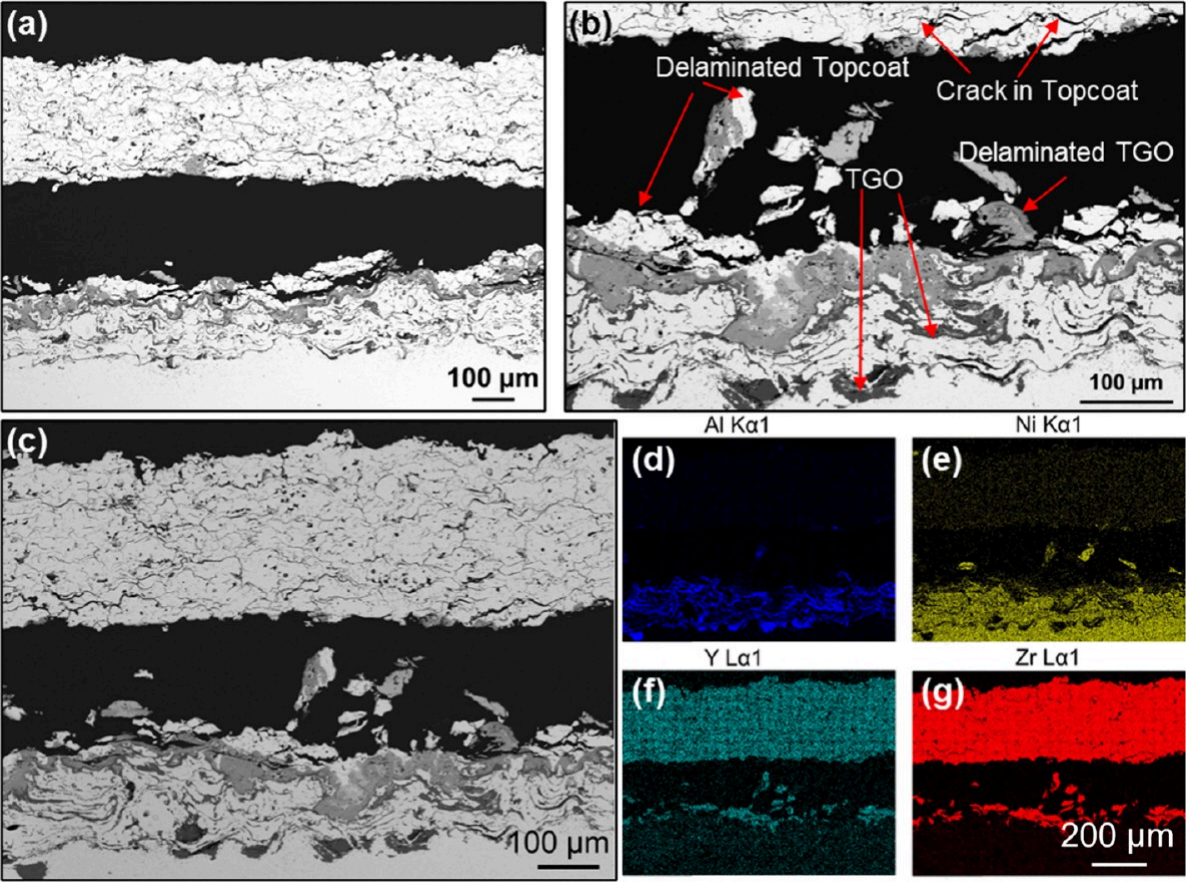
APS YSZ TBC FCT failure; (a) cross-sectional micrographs of the failed APS coatings post 0.3 N_T_ thermal cycles, (b) zoomed region showings cracks and delamination of the topcoat and the bond coat, (c) EDS mapping region and corresponding element maps of (d) aluminum, (e) nickel, (f) yttrium, and (g) zirconium.

The failure in APS coatings was abrupt, with the entire coating spalling, unlike that observed with SPS and EBPVD coatings. These coatings failed comparatively earlier than those of SPS and EBPVD coatings. The coating failure was observed through cohesive cracks within the topcoat, in the TGO, as well as at the interface between the TGO-topcoat and TGO-bond coat. It was difficult to measure the average TGO thickness in this case, as the oxides were found to be present in the bond coat porosity. Apart from aluminum oxide, some amount of chromium aluminum spinel oxide also formed during the thermal cycles (darker regions in Figure 6c as evident from the Cr elemental mapping shown in Figure S2). Phase transformations were not evident in the APS coatings after thermal cycling failure.

### 3.3 CMAS Exposure Tests

The samples exposed to CMAS showed significant penetration of CMAS melts into the coatings. Two different coatings (for each deposition technique) were exposed to the CMAS tests. The digital images of the coatings after CMAS exposure are shown in Figure S3. It was evident that there was some partial delamination of some of the coatings along the edges as a result of the CMAS thermal exposure. The behavior of each coating against CMAS exposure is explained as follows:

#### 3.3.1 SPS CMAS Failure

The cross-sectional BSE micrographs with Ca, Mg, Al, and Si elemental maps and the Raman spectra of the SPS coatings exposed to CMAS corrosion are listed in Figure 7. On thermal exposure, the infiltration of CMAS melts can be observed through the vertical columnar gaps into the SPS topcoat. The solidified residual glassy CMAS can also be seen at the top of the coatings. Significant TGO formation was identified at the topcoat-bond coat interface, which is attributed to the oxidation of the bond coat upon heat treatment. The elemental mapping showed that some amounts of Ca, Mg, and Si were also present at the topcoat-TGO interface, which implies full penetration of CMAS melts into the SPS topcoat. Raman analyses of the CMAS exposure SPS coatings showed that there was considerable monoclinic phase formation (indicated by points 2 and 3 in the mapped Raman area) at the top surface of the coatings, which is in direct contact with the CMAS melt. Although there was significant penetration of CMAS melts, no signs of monoclinic phase formation were observed in the regions away from the top surface (point 1). This could be because of the limited presence of CMAS melt that is insufficient to cause the formation of yttria-depleted monoclinic zirconia through YSZ dissolution and resolidification in the SPS columnar gaps.

**Figure 7:**
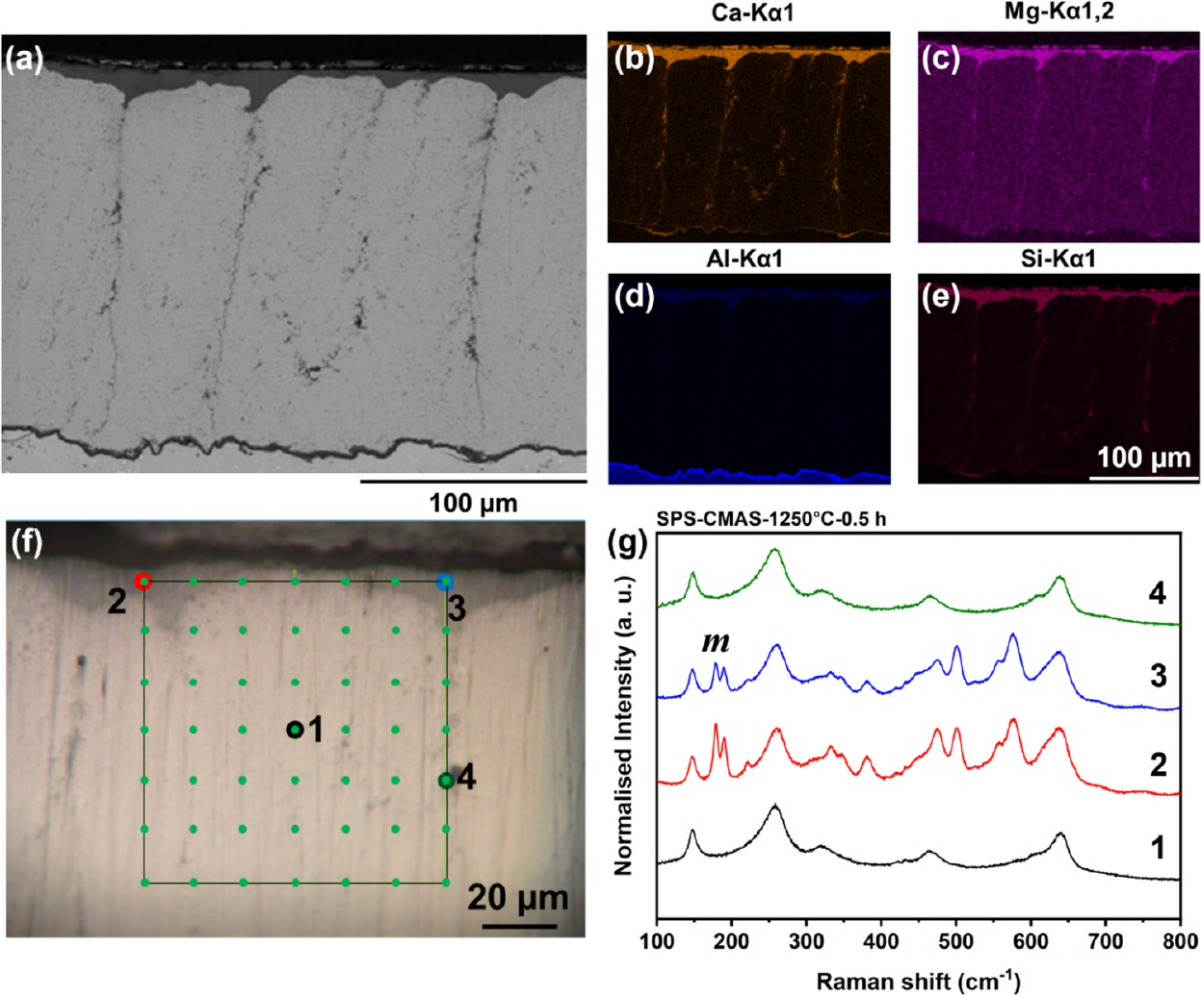
SPS YSZ TBC CMAS failure; (a) cross-sectional micrograph showing CMAS infiltration into the SPS topcoat and the corresponding EDS elemental maps for (b) calcium, (c) magnesium, (d) aluminum, (e) silicon, (f) Raman mapping area, and (g) Raman spectra of three different points of interest.

#### 3.3.2 EBPVD CMAS Failure

The cross-sectional BSE micrographs with Ca, Mg, Al, and Si elemental maps and the Raman spectra of the EBPVD coatings exposed to CMAS corrosion are listed in Figure 8. The molten CMAS was observed to penetrate fully into the EBPVD topcoat. The extent of infiltration depends upon the width of the columnar gaps. Broader columnar gaps will ease the pathways for the CMAS to penetrate, while the capillary pressure and the frictional drag of the contact surfaces primarily govern the CMAS infiltration in the narrow channels. CMAS residues were observed on the top surface of the topcoat. The penetration of CMAS was found to be greater compared to SPS owing to a higher amount of Ca, Mg, and Si elements observed at the interface of the topcoat and the bondcoat. Similar to the behavior of SPS coatings against CMAS corrosion, the monoclinic phase was evident at the top surface but was not observed in the regions away from the top surface.

**Figure 8:**
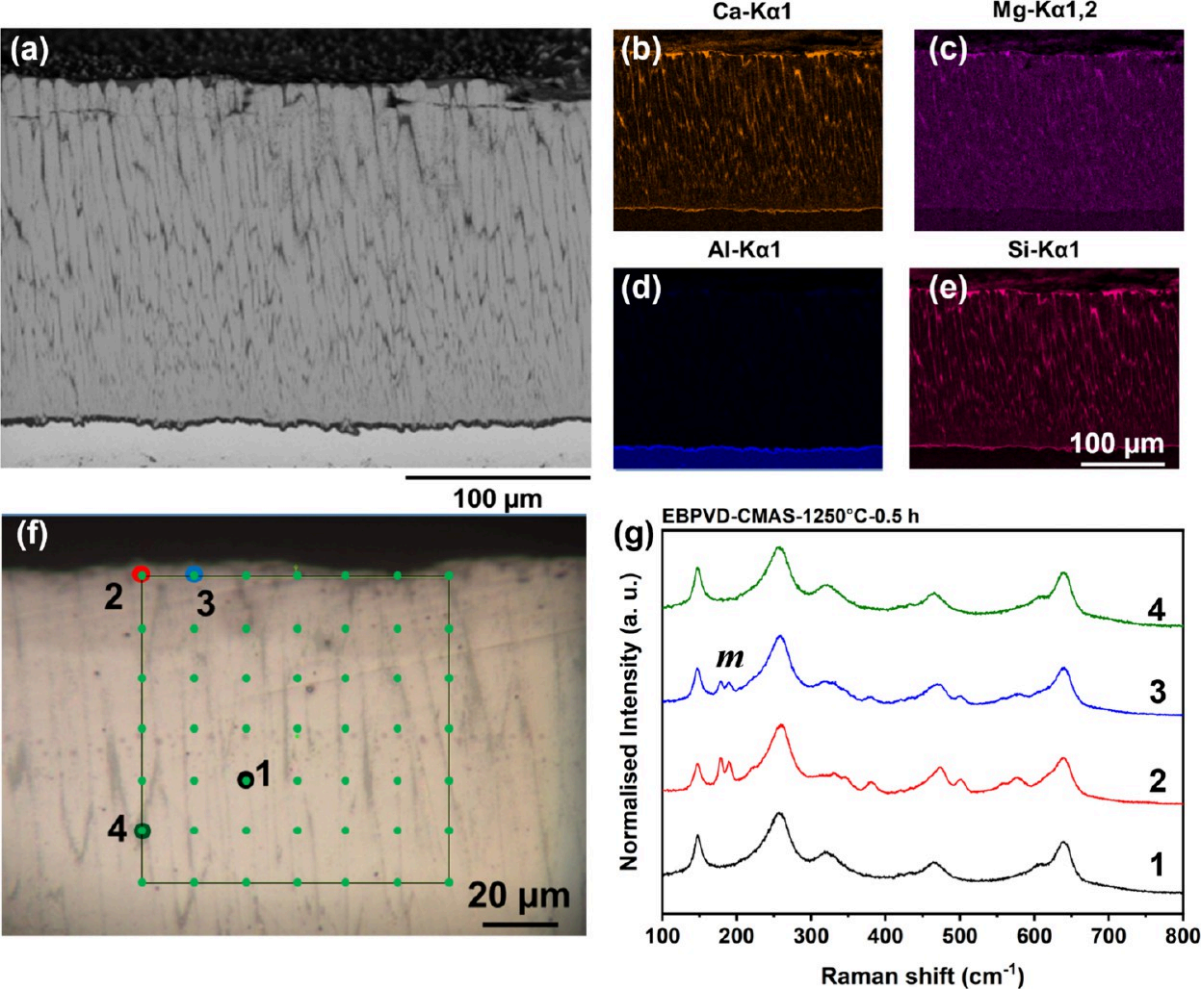
EBPVD YSZ TBC CMAS failure; (a) cross-sectional micrograph showing CMAS infiltration into the EBPVD topcoat and the corresponding EDS elemental maps for (b) calcium, (c) magnesium, (d) aluminum, (e) silicon, (f) Raman mapping area, and (g) Raman spectra of three different points of interest.

#### 3.3.3 APS CMAS Failure

The cross-sectional BSE micrographs with Ca, Mg, Al, and Si elemental maps and the Raman spectra of the APS coatings exposed to CMAS corrosion are shown in Figure 9. Unlike SPS and EBPVD coatings, CMAS residues were not observed at the top surface of the coatings. This is because the thickness of the APS topcoat was comparatively higher, and CMAS was observed to be infiltrated into the whole coating depth. Also, CMAS tends to chemically react with APS YSZ topcoat owing to the penetration of Ca, Mg, Al, and Si through open pores, cracks, and intersplat boundaries and form Ca/Zr crystalline products as well as other crystalline silicates instead of leaving a glassy residue at the top.^49–52^ The advantage of APS coatings is that there are no vertical channels that provide the pathway for CMAS to penetrate the topcoat; however, open pores, cracks, and intersplat boundaries do provide a pathway for the CMAS melt to penetrate, leading to full infiltration. On the contrary, Raman analyses showed some regions within the topcoat away from the top surface that contained some amount of monoclinic phase as a result of the dissolution of YSZ into the CMAS melts and reprecipitation of yttria-depleted monoclinic zirconia. It must be noted that the monoclinic phase was not evident in the topcoat away from the surface in the case of SPS and EBPVD YSZ coatings. Similar to SPS and EBPVD coatings, monoclinic phase formation was also observed at the top surface of the coatings, and some regions away from the top surface showed no evidence of tetragonal-to-monoclinic phase transitions.

**Figure 9:**
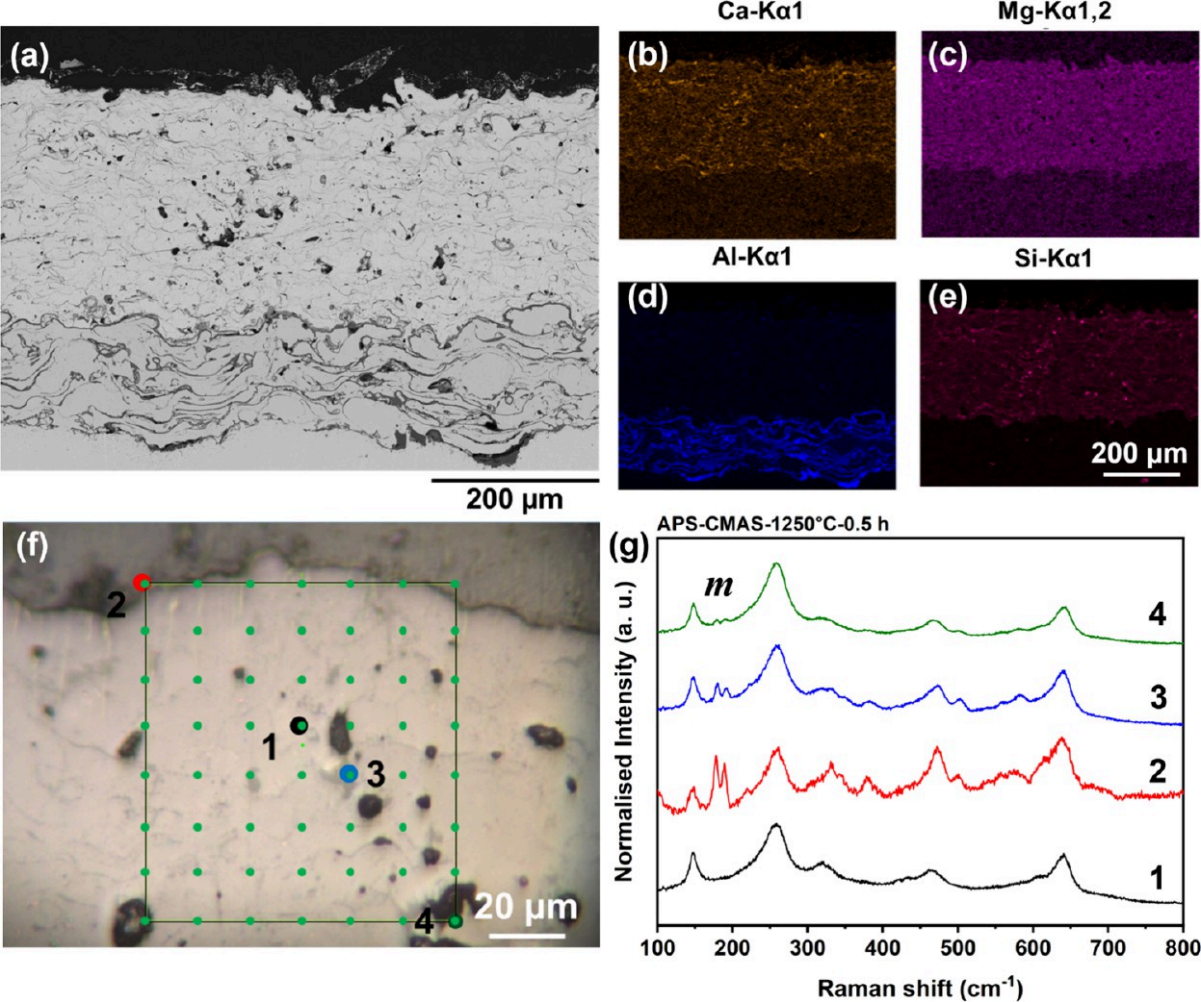
APS YSZ TBC CMAS failure; (a) cross-sectional micrograph showing CMAS infiltration into the APS topcoat and the corresponding EDS elemental maps for (b) calcium, (c) magnesium, (d) aluminum, (e) silicon, (f) Raman mapping area, and (g) Raman spectra of three different points of interest.

## 4 Discussion

### 4.1 Thermal Cycle Fatigue Failure in YSZ Coatings

There are various mechanisms involved during thermal cycling that can lead to TBC failure.^53^ One of these failures occurs due to the thermal stresses in various layers arising from the coefficient of thermal expansion mismatch between the coatings, TGO, bond coat, and the substrate. Another prominent phenomenon is the stress due to the growth of the TGO layer, which leads to stress accumulation resulting in coating spallation. The thermally grown oxide (TGO) layer forms during each thermal cycle. This layer grows with increasing thermal cycles due to the diffusion of aluminum toward the YSZ-bondcoat interface.^54^ Mahade et al. proposed that the TGO thickness beyond which TBC spallation takes place is 5 μm.^25,48^ In addition to this, surface rumpling/ratcheting of the bond coat as a result of cyclic oxidation gives rise to cracks at the TGO-topcoat interface, which then coalesce to form larger cracks sufficient to cause delamination failure of the topcoat.^11,55,56^ This phenomenon was more prominent in SPS and EBPVD coatings with PtAl bond coatings.

The underlying mechanism for the failure of SPS coatings can thus be explained as follows:

1. The stresses arise due to the mismatch between the thermal expansion coefficients of the various layers. The growth of the TGO thickness with subsequent thermal cycles results in a decrease in fracture toughness and accumulation of internal stresses in the topcoat.
2. Microcracks within the TGO, which could have originated from the TGO-bondcoat interface and extended toward the coating. This led to the detachment of TGO as well as the topcoat after thermal cycling.
3. Significant sintering and the growth of vertical columnar cracks were observed, which also contributed to TBC failure after thermal cycling.
4. The phase transformation from tetragonal-to-monoclinic YSZ was not observed by using XRD after thermal cycling (Figure 2).

The initiation and propagation of coating cracks in the EBPVD coatings are mainly because of the TGO growth stress, and the elastic stresses developed by repeated thermal shocks and high-speed cooling gas impact. In case of APS coatings, the entire coating delaminated, and the failure was mainly observed within the topcoat, in the TGO, as well as at the interface between TGO-topcoat and TGO-bond coat. It must be noted that the bondcoat in the APS case does not have as good oxidation performance as the one used for EBPVD and SPS, negatively impacting the lifetime. Since we cannot deposit an APS coating directly on the diffusion bond coat owing to the smoother surface of the Pt–Al bond coat, it is not possible to make a direct comparison. The oxidation resistance of platinum aluminide (Pt–Al) bond coats, used with SPS and EBPVD coatings in the present study, is superior to that of CoNiCrAlY systems employed in APS coatings. This is primarily due to their ability to rapidly form a continuous and slow-growing alpha alumina (Al_2_O_3_) scale. The presence of Pt enhances selective Al activity and outward Al diffusion, facilitating early establishment of a protective alumina layer while suppressing transient oxide formation. In contrast, CoNiCrAlY coatings initially form mixed oxides such as NiO, Cr_2_O_3_, and spinel phases before transitioning to a more stable alpha alumina-dominated scale, resulting in higher initial oxidation rates. Furthermore, Pt–Al systems exhibit improved thermally grown oxide (TGO) stability and reduced interfacial rumpling under cyclic conditions, whereas CoNiCrAlY coatings are more prone to nonuniform oxide growth and stress accumulation.^57^

#### Comparison of Furnace Cycling Failure

The SPS YSZ coatings were found to have the highest FCT lifetime as compared with EBPVD samples and APS samples. The comparison of the lifetime of various coatings against thermal cycling is shown by utilizing a bar chart (Figure 10) and Weibull distribution plot^58,59^ (Figure S4).

**Figure 10:**
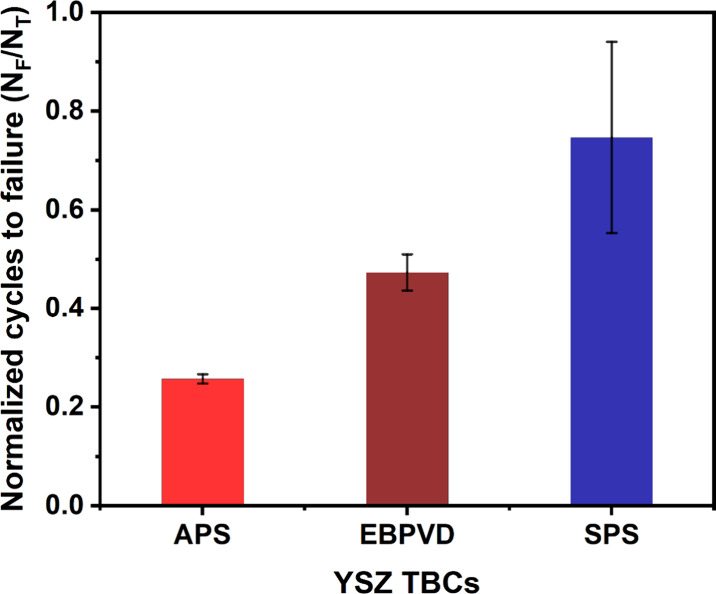
Normalized cycles to failure for SPS, EBPVD, and APS YSZ TBCs.

The substantial difference in thermal cycling lifetime between SPS and EBPVD coatings can be attributed to the larger column diameter (54 ± 7 μm) in SPS coatings as compared to EBPVD coatings (6 ± 0.5 μm), which limits the horizontal propagation of delamination cracks between adjacent intercolumnar gaps. The lower density of intercolumnar gaps in SPS coatings may reduce the probability of crack initiation and subsequent linkage between adjacent gaps, thereby limiting crack propagation during thermal cycling, which further leads to TBC spallation. The larger columnar diameter and subsequently increased gaps between vertical columnar gaps in the case of SPS coatings, as compared to EBPVD coatings, reduce the tendency for the cracks to propagate and bridge those vertical gaps (which subsequently leads to spallation), as cracks need to travel a larger distance.

### 4.2 CMAS Failure in YSZ Coatings

Another failure mechanism in TBCs includes the ingestion of CMAS deposits, followed by their subsequent melting and infiltration upon reaching high temperature during service.^60^ This may arise due to the melting of silicate deposits (in the form of runway debris, sand, fly/volcanic ash, dust, etc.) at high operating temperature (melting/softening point of CMAS ≈ 1190–1260 °C). The infiltration of CMAS into pores, microcracks, and defects in the topcoat affects the strain tolerance of TBCs. It induces additional stresses upon cooling owing to a large CTE mismatch with 7YSZ TBCs. In addition to this, the destabilization of *t′-*YSZ by the corrosive attack of molten CMAS deposits may cause premature delamination of the TBCs and induce local hotspots, causing the accelerated degradation of the underlying metallic components when exposed directly to extremely hot gases.^34^ On CMAS infiltration, YSZ dissolves into CMAS melts, and on cooling, reprecipitation of monoclinic zirconia takes place, which is detrimental for TBCs. CMAS infiltration into the coatings increases the stiffness, reduces strain tolerance, and also affects the porosity as well as the overall microstructure.^50,51^ It was observed that all the coatings were fully infiltrated by CMAS; however, the extent of the infiltration was comparatively dissimilar. CMAS penetration percentage was calculated using ImageJ software in each of these coatings, as shown in Table 2. For this, the thresholding technique was used while eliminating the glassy layer at the top surface of the coatings to obtain the percentage of CMAS penetrated into the coatings. While SPS coatings had CMAS penetration of only ∼2.1%, EBPVD and APS coatings showed significant CMAS infiltration of ∼9.1% and ∼7.4%, respectively. The tetragonal-to-monoclinic transformation was observed on the top surface in all the coatings, and some regions away from the top surface only in the case of APS coatings. This may be attributed to the higher local concentration of CMAS in the region away from the top surface owing to capillary-driven infiltration of molten CMAS into the bulk through interconnected porosity and microcracks, which promotes reactions with the APS YSZ coating, leading to the formation of monoclinic zirconia. Figure 11 shows a schematic representation summarizing the failure mechanisms observed against FCT and CMAS corrosion in SPS, EBPVD, and APS YSZ TBCs.

**Table 2:** CMAS Penetration (%) in SPS, EBPVD, and APS YSZ Coatings

YSZ coating	CMAS penetration (area %)
SPS	∼2.1%
EBPVD	∼9.1%
APS	∼7.4%

**Figure 11:**
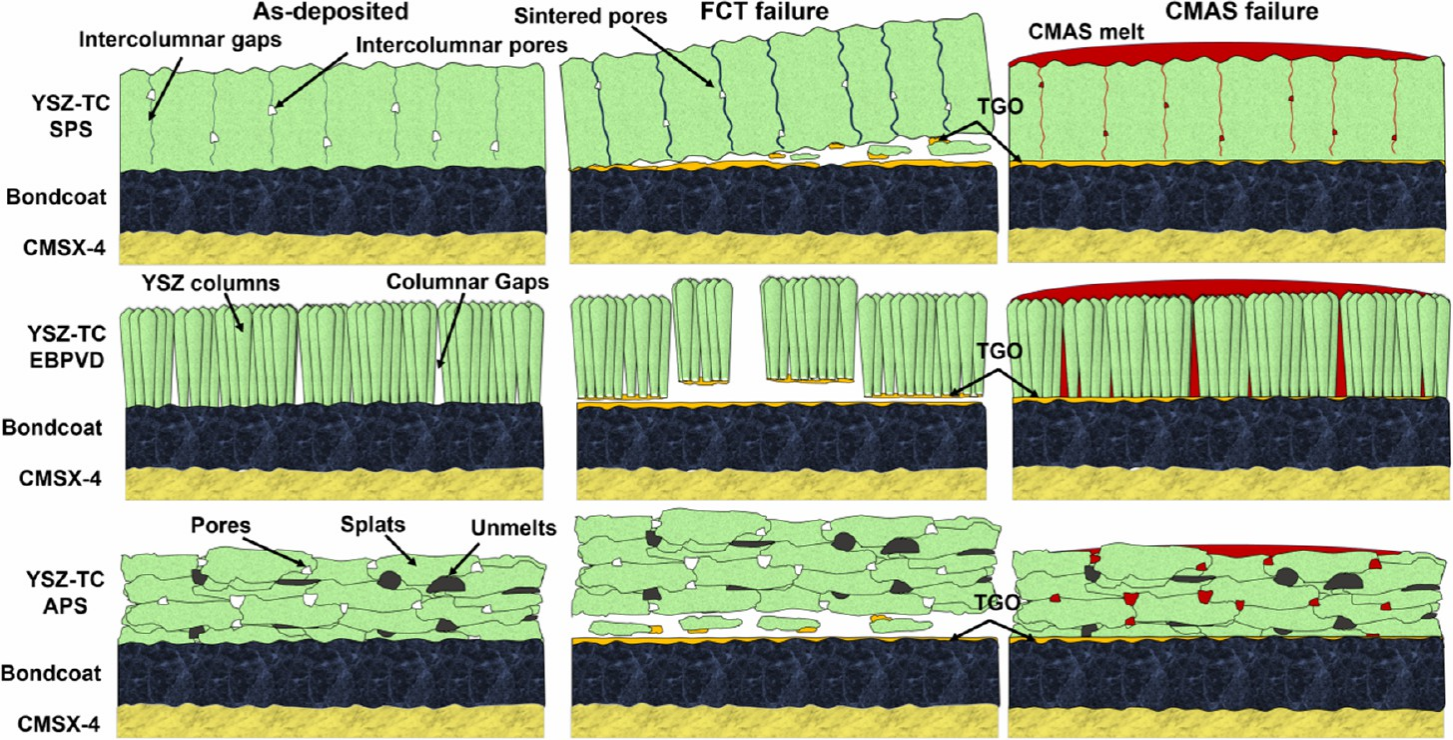
Schematic representation of the FCT and CMAS failure mechanisms in SPS, EBPVD, and APS YSZ coatings.

The difference in the CMAS penetration between SPS and EBPVD coatings can be attributed to the differences in microstructural features within the coatings, such as column density (number of columns per unit length in the cross section) and column diameter. EBPVD coatings contain a higher column density (163 ± 13 columns/mm) compared to SPS (19 ± 3 columns/mm), providing more continuous capillary pathways for CMAS infiltration. In addition to this, the gap between feathery arms can also act as additional capillary channels in EBPVD coatings, allowing CMAS to infiltrate through them.^28^ Conversely, the wider columns and lower column density in SPS coatings lead to fewer intercolumnar channels, thereby restricting CMAS penetration. This structural difference likely explains the comparatively lower CMAS infiltration observed in SPS coatings. Additionally, as mentioned earlier, APS coatings typically contain open and interconnected porosity, which facilitates CMAS infiltration. The presence of relatively larger defects within the coating increases the local volume of infiltrated CMAS, promoting enhanced interaction with YSZ and consequently accelerating the formation of monoclinic zirconia. This process involves dissolution of yttria (and to some extent, zirconia) into the CMAS melt, leading to destabilization of the tetragonal phase and subsequent formation of monoclinic zirconia upon cooling.

The columnar gap in SPS and EBPVD coatings was measured to be 1.5 ± 0.7 μm and 0.6 ± 0.07 μm, respectively; however, despite the smaller gap size, the higher columnar density in EBPVD coatings promotes greater CMAS infiltration compared to SPS, as evidenced by area fraction analysis. Consequently, a larger volume of CMAS is accommodated within the EBPVD coating, whereas in SPS coatings, a greater fraction remains at the top surface. These observations indicate that CMAS-induced failure is governed not only by the size of open channel gaps but also by the overall infiltration pathways within the coating and the effective interaction volume of CMAS, which promotes monoclinic phase formation due to yttria depletion from YSZ.

## 5 Conclusions

For the first time in the published literature, a scientific study was undertaken to deposit an SPS columnar structure from YSZ suspension on a Pt–Al bond-coated CMSX-4 single-crystal nickel substrate. This study provides a systematic and direct comparison of YSZ coatings produced via SPS, EBPVD, and APS, with each optimized to yield representative microstructures. All coatings were deposited on CMSX-4 single-crystal Ni-based superalloy substrates and evaluated under identical thermal cycling and CMAS exposure conditions, enabling a rigorous and meaningful assessment. APS coatings, considered a baseline, exhibited the lowest thermal cycling lifetime. In contrast, SPS coatings showed superior performance, outperforming both APS and EBPVD coatings and exhibiting the highest thermal cycling durability. No phase transformations were observed in any coating following the thermal cycling failure. Under CMAS exposure, full penetration and monoclinic phase formation were observed primarily near the top surface of the coatings. CMAS penetration was most pronounced in EBPVD coatings, followed by APS and SPS coatings; however, APS coatings exhibited localized monoclinic phase formation beneath the surface, which was not observed in the EBPVD and SPS coatings. Overall, under identical test conditions in the present study, SPS coatings with optimized parameters demonstrate promising potential and performance compared to EBPVD and APS coatings.

## Supplementary Material



## Data Availability

The data sets generated during and/or analyzed during the current study are available from the corresponding author on reasonable request.

## Notes

(Figure S1) Camera view of specimens exposed to thermal cycling; (Figure S2) evidence of the formation of spinel oxide containing chromium and aluminum, along with Al_2_O_3_ in the FCT failed APS YSZ coatings; (Figure S3) digital images of SPS, EBPVD, and APS YSZ coatings after CMAS exposure; (Figure S4) Weibull distribution plot for comparing FCT lifetimes of SPS, EBPVD, and APS YSZ TBCs; (Table S1) normalized cycles to failure for SPS, EBPVD, and APS coatings; and (Table S2) Weibull distribution plot parameters (PDF)
