# Supplementary material for: Yttria-Stabilized Zirconia Deposited Using Suspension Plasma Spraying: A Comparative Framework with Electron Beam Physical Vapor Deposition and Air Plasma Spraying
Source: ACS Appl Mater Interfaces. 2026 Jun 12;18(24):34222–34. doi: 10.1021/acsami.6c05211 (PMC13307067; doi:10.1021/acsami.6c05211)
Supplement: Supplementary file 1 [file am6c05211_si_001.pdf]

Supporting information

Yttria stabilized zirconia deposited using suspension plasma spraying: a comparative framework with electron beam physical vapour deposition and air plasma spraying

Vikram Hastak[1*], Kah Leng[1], Siddharth Lokachari[1], Nicholas Curry[1, 2], Gyaneshwara Brewster[3], Andy Norton[3], Tanvir Hussain[1*]

[1]*Centre of Excellence in Coatings and Surface Engineering, Faculty of Engineering, University of Nottingham, Nottingham NG7 2RD, UK*

[2]*Thermal Spray Innovations, Salzburg 5662, Austria*

[3]*Rolls-Royce Plc, Derby DE24 8BJ, UK*

**Corresponding Authors: Vikram.Hastak@nottingham.ac.uk*

Tanvir.Hussain@nottingham.ac.uk

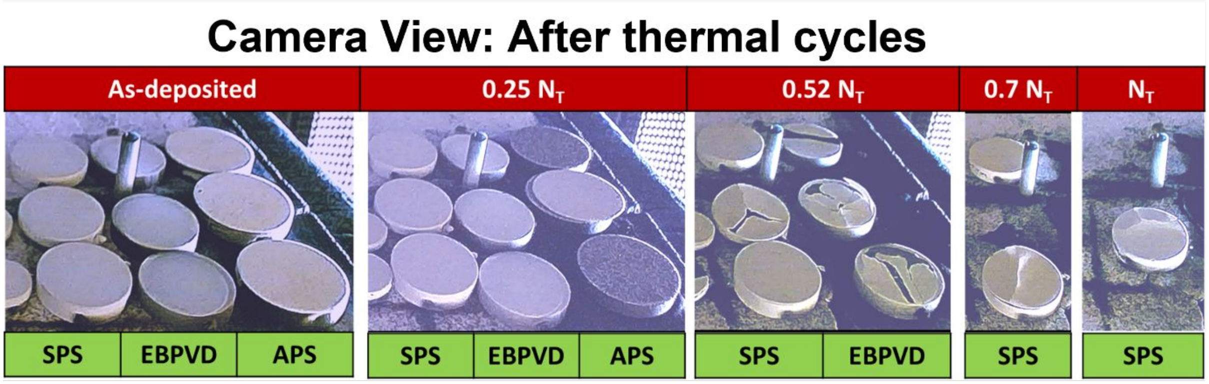

Figure S1 *Camera view of specimens exposed to thermal cycling. N_T is the maximum number of thermal cycles.*

Table S1 *Normalized cycles to failure for SPS, EBPVD and APS coatings. N_F is the number of cycles to failure, and N_T is the maximum number of thermal cycles*

**Sample Code**	**FCT Lifetime (Normalized cycles to failure; N_F/N_T)**		
	Sample 1	Sample 2	Sample 3
**SPS**	1	0.53	0.71
**EBPVD**	0.47	0.52	0.43
**APS**	0.25	0.25	0.27

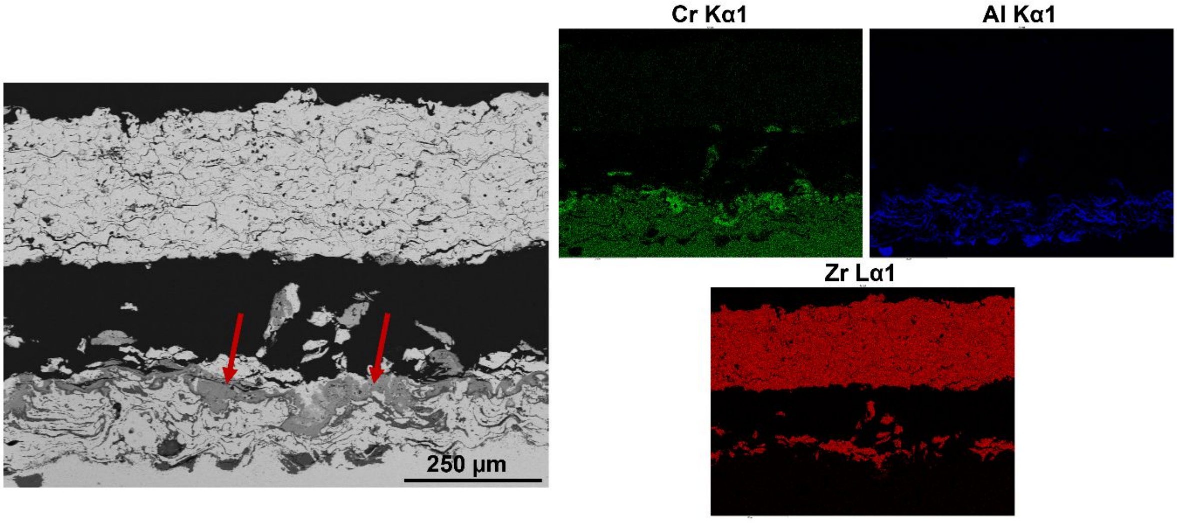

Figure S2 *Evidence of the formation of spinel oxide containing chromium, aluminium along with Al_2O_3 in the FCT failed APS YSZ coatings*

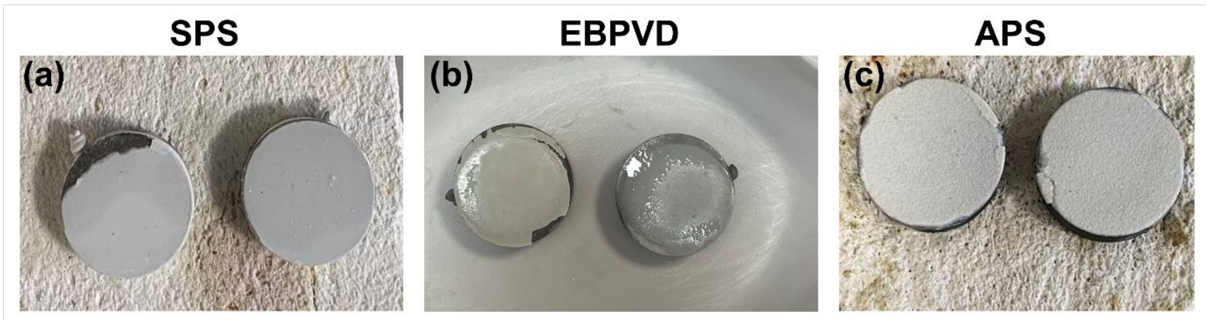

Figure S3 *Digital images of (a) SPS, (b) EBPVD and (c) APS YSZ coatings after CMAS exposure*

Comparison of FCT lifetimes using Weibull distribution plots:

The Weibull plots gave a good linear correlation for the normalized cycles to failure in EBPVD and SPS ($R^2 = 0.99$) coatings.

Weibull function:

$$F(t) = 1 - e^{-\left(\frac{t}{\alpha}\right)^{\beta}} \quad (1)$$

Simplified equation:

$$\ln\left(\ln\left(\frac{1}{1-F(t)}\right)\right) = \beta lnt - (2)$$

Here, $F(t)$ is the cumulative probability of failure and t is the normalized cycles to failure. β is the shape parameter, which is related to the scatter of the data. The higher the value of β, the smaller the scatter. α is the scale parameter, which represents the normalized cycles to failure of 63.2% of the samples. **Table S2** shows the parameters obtained from the Weibull distribution plots of the normalized cycles to failure in SPS, EBPVD, and APS YSZ coatings. While the data for SPS showed lower reliability than APS and EBPVD, the mean normalized thermal cycle lifetime was the highest for YSZ coatings deposited using SPS.

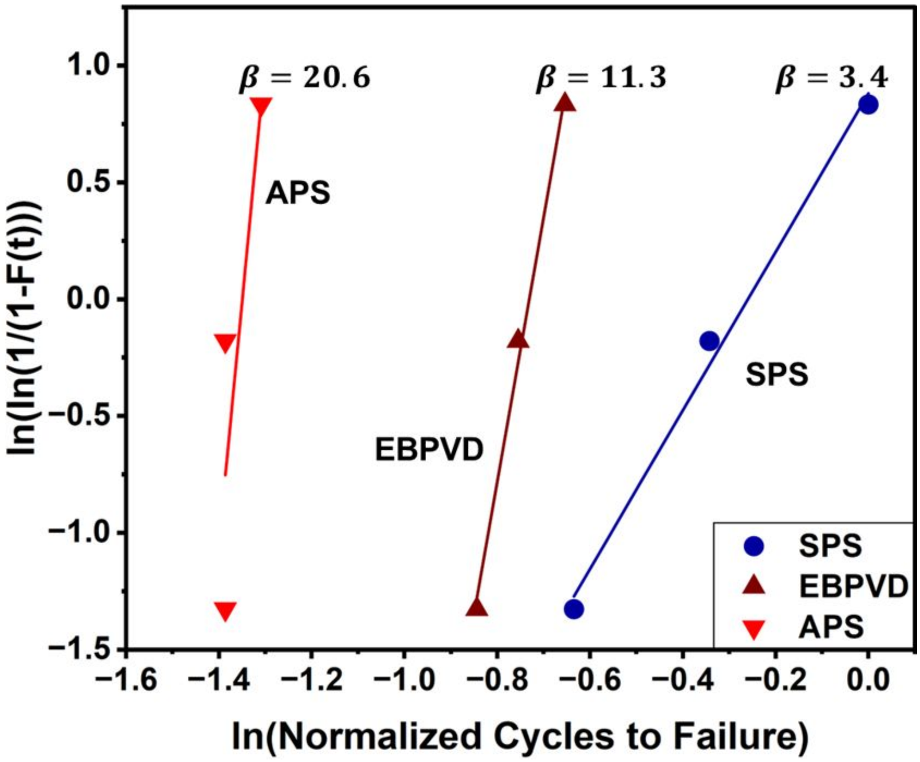

Figure S4 *Weibull distribution plot for comparing FCT lifetimes of SPS, EBPVD and APS YSZ TBCs.*

Table S2 *Weibull distribution plot parameters*

Sample	β	α (Normalized in terms of N_T)	R^2
**SPS**	3.4	0.77	0.99
**EBPVD**	11.3	0.48	0.99
**APS**	20.6	0.26	0.71
